# Synthesis and crystal structures of 6- and 8-methyl-3-phenyl­benzo[*e*][1,2,4]triazines

**DOI:** 10.1107/S2056989025005882

**Published:** 2025-07-17

**Authors:** Christos P. Constantinides, Syed Raza, Fadwat Bazzi, Nisreen Sharara, Simona Marincean

**Affiliations:** a University of Michigan-Dearborn, 4901 Evergreen Rd., Dearborn, Michigan 48128, USA; University of Missouri-Columbia, USA

**Keywords:** benzo[*e*][1,2,4]triazine, π–π stacking, aza-rich heterocycles, triazines, crystal structure

## Abstract

The synthesis and single-crystal X-ray structures of two regioisomeric Blatter radical precursors, 6-methyl-3-phenyl­benzo[*e*][1,2,4]triazine, (**I**), and 8-methyl-3-phenyl­benzo[*e*][1,2,4]triazine (**II**), C_14_H_11_N_3_, are reported. Both compounds feature planar heteroaromatic frameworks with extensive π-conjugation across the benzo[*e*][1,2,4]triazine core. Compound **I** crystallizes in the ortho­rhom­bic space group *Pbca*, while **II** adopts the monoclinic space group *P*2_1_/*c*. Structural analysis reveals nearly identical bond lengths and angles across both isomers, indicating minimal influence of methyl substitution on core geometry. Supra­molecular features are dominated by π–π stacking inter­actions, leading to one-dimensional columnar arrangements. Compound **I** exhibits alternating dimers with slippage in both stacking directions and forms hydrogen-bonded chains that generate dense, wave-like sheets. Compound **II** displays uniform stacking with regular inter­planar distances and pronounced translational overlap. These structural insights contribute to understanding how regioisomeric substitution patterns influence the solid-state organization of Blatter radical precursors, with implications for their application in mol­ecular electronics and spin materials.

## Abstract 2

The crystal structures of two Blatter radical precursors, 6-methyl-3-phenyl­benzo[*e*][1,2,4]triazine and 8-methyl-3-phenyl­benzo[*e*][1,2,4]triazine, exhibit extended conjugated heteroaromatic frameworks, with unidimensional columnar arrangements governed by π–π stacking inter­actions with little impact from the methyl substituent position on the core geometry.

## 1. Chemical context

A brief literature survey on benzo[*e*][1,2,4]triazine and its derivatives reveals more than 50,000 publications in the last decade. This class of compounds garnered the inter­est of the research community (Mohammadi Ziarani *et al.*, 2019 ▸; Bodzioch *et al.*, 2019 ▸) due to their biological activity and unique structural characteristics that led to a wide range of applications in medicinal chemistry and material science. From a pharmacological perspective they have been found to exhibit anti­cancer (Shi *et al.*, 2018 ▸; Qi *et al.*, 2022 ▸), anti­tumor (Noronha *et al.*, 2006 ▸, 2007 ▸; Cascioferro *et al.*, 2017 ▸; Keane *et al.*, 2018 ▸), anti­bacterial (Sztanke *et al.*, 2007 ▸; Arshad *et al.*, 2017 ▸) anti­malaria (Wolf *et al.*, 1954 ▸; Pchalek *et al.*, 2006 ▸), anti-inflammatory (Gao *et al.*, 2015 ▸), anti­viral (Kotovskaya *et al.*, 2007 ▸), and anti­proliferative (Sparatore & Sparatore, 2024 ▸) activities. This versatile structure–activity relationship has made benzo[*e*][1,2,4]triazine an important scaffold in drug development and several synthetic protocols (Nematpour & Nouri, 2025 ▸). At the same time benzo[*e*][1,2,4]triazine deriv­atives are precursors to the well-known Blatter radicals recognized for their air, thermal and moisture stability (Rogers *et al.*, 2020 ▸; Constanti­nides & Koutentis, 2016 ▸). Given the sensitivity of the overall behavior of the radicals to the structural changes induced by derivatization of the benzo[*e*][1,2,4]triazine skeleton, the scope of their applications in the field of magnetic materials has expanded tremendously in recent years to encompass sensors, liquid crystals, and spin labels. We report here the synthesis and crystal structure of two Blatter radical precursors: 3-phenyl-6-methyl­benzo[*e*][1,2,4]triazine (**I**) and 3-phenyl-8-methyl­benzo[*e*][1,2,4]triazine (**II**). For the structural parameters of the reported structures, we will use the crystallographic numbering rather than the nomenclature-based one.
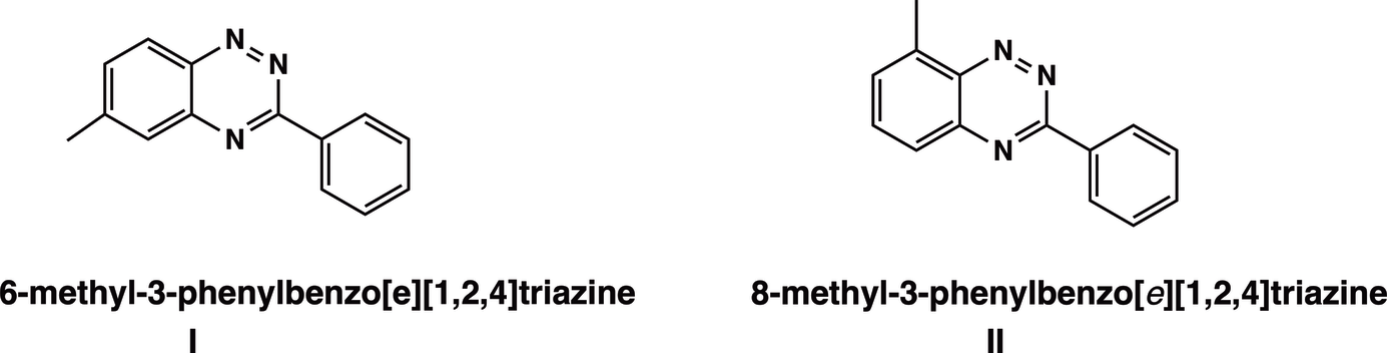


## 2. Structural commentary

**Figure 1:**
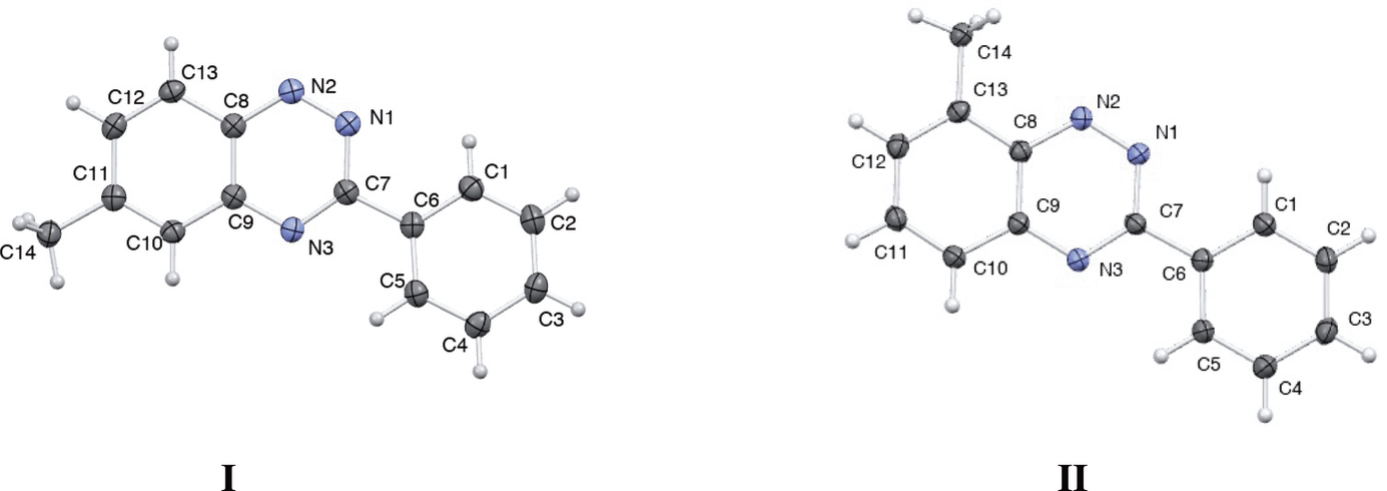
A view of the mol­ecular structures of **I** and **II** with crystallographic atom labeling and displacement ellipsoids drawn at the 50% probability level.

Suitable crystals of the title compounds for single-crystal X-ray diffraction were obtained by slow cooling of concentrated ethanol solutions. Compound **I** crystallizes in the ortho­rhom­bic space group *Pbca*, with one mol­ecule in the asymmetric unit and eight mol­ecules in the unit cell. Compound **II** crystallizes in the monoclinic space group *P*2_1_/*c*, with one mol­ecule in the asymmetric unit and four mol­ecules in the unit cell. The mol­ecular structures of **I** and **II** (Fig. 1 ▸) are essentially planar with the angle between the benzotriazine core and the 3-phenyl substituent at 9.68 (4)° and 4.37 (5)° for **I** and **II**, respectively. Selected bond lengths for mol­ecules **I** and **II** are presented in Table 1 ▸. The bond distances within the triazine ring, namely C7—N1, N1—N2, N2—C8, C7—N3, and C9—N3, range from 1.311 to 1.374 Å and are consistent with a delocalized heteroaromatic system. These values lie inter­mediate between typical C–N and N–N single and double bonds, reflecting extensive π-conjugation within the six-membered triazine ring.

Slight elongation is observed in the C—C bonds flanking the triazine moiety, particularly C8—C9 and C13—C8 [1.421 (2)–1.422 (2) Å] compared to the more uniform bond lengths of the adjacent benzene ring [*e.g*., C10—C11 and C12—C13, 1.362 (2)–1.370 (2) Å]. This bond elongation is likely a consequence of the electron-withdrawing nature of the adjacent nitro­gen atoms, which may diminish π-overlap and increase single-bond character in these positions. The remainder of the aromatic framework displays modest bond length alternation, consistent with partial localization of π-electrons while maintaining overall aromaticity. Notably, the near-identical bond lengths observed between mol­ecules **I** and **II** indicate a high degree of structural similarity in the solid state, suggesting that crystal packing exerts minimal influence on the electronic structure of the triazine core.

The bond angles in **I** and **II** reveal a largely planar, conjugated structure consistent with aromatic delocalization. Angles within the triazine ring, such as C8—N2—N1 and N2—N1—C7, are close to 119.1 (1)° in both mol­ecules, reflecting typical *sp*^2^ hybridization and a delocalized electronic structure. The N1—C7—N3 angle is slightly widened [125.9 (1)°], likely due to conjugation and geometric constraints from ring fusion, while the C7—N3—C9 angle is correspondingly compressed [115.8 (9)°], as expected for fused aromatic systems. Angles around the fused aryl ring, including N3—C9—C8, C9—C8—N2, and C13—C8—C9, remain close to 120°, with only minor variations between the two mol­ecules. Slight shifts, such as the increase of the C13—C8—C9 angle from 119.7 (1)° to 121.1 (1)°, are balanced by decreases elsewhere [*e.g.*, C12—C13—C8 from 119.2 (1)° to 117.2 (1)°], suggesting subtle conformational differences likely due to crystal packing. In the peripheral phenyl ring, bond angles deviate slightly from the ideal 120° but remain within the range expected for aromatic systems. The C9—C10—C11 and C10—C11—C12 angles shift modestly between the two mol­ecules, consistent with minor local distortions rather than electronic differences. Overall, the bond angles confirm a planar, delocalized structure with excellent agreement between **I** and **II**, highlighting the rigidity and symmetry of the benzo[*e*][1,2,4]triazine framework.

## 3. Supra­molecular features

**Figure 2:**
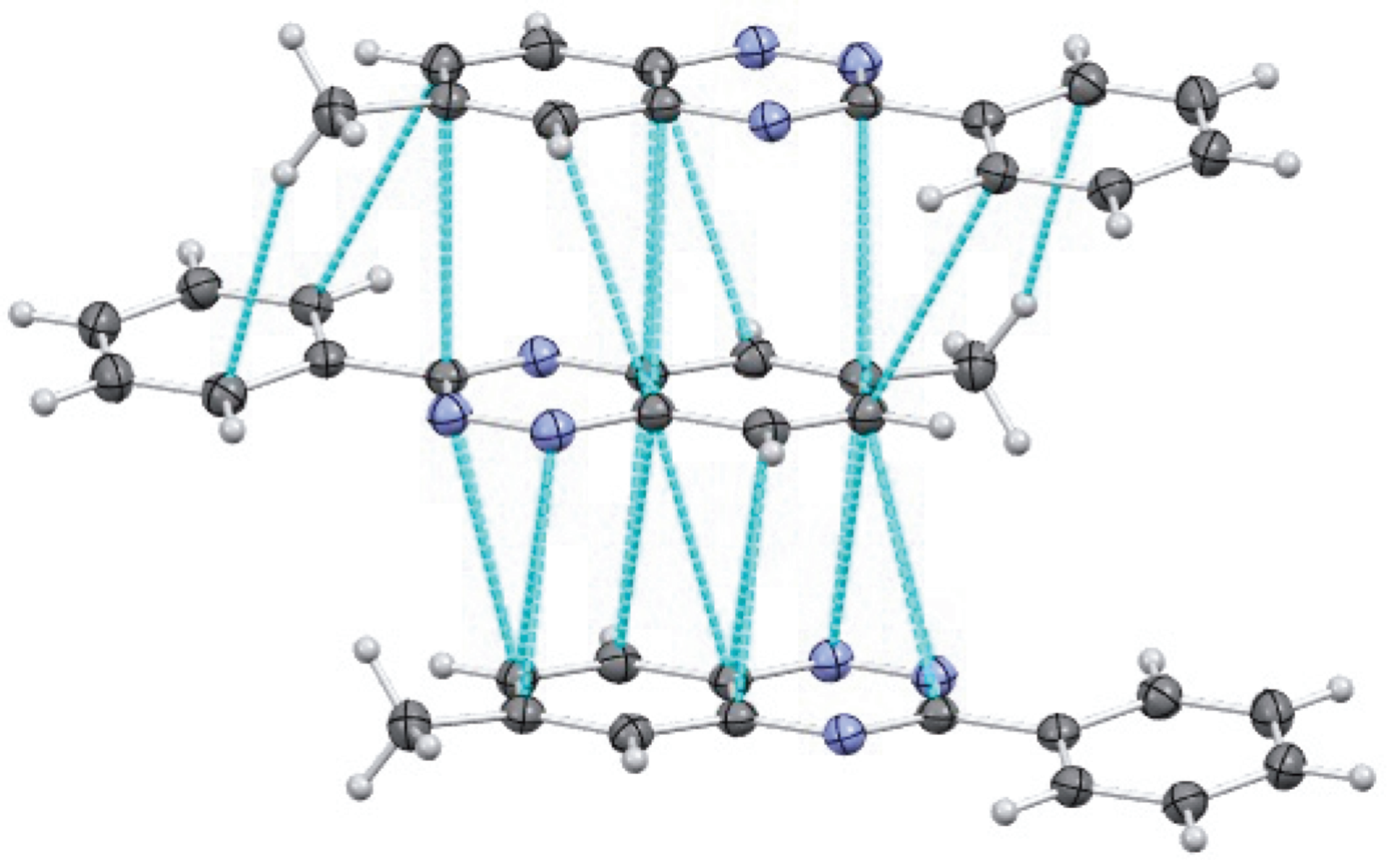
One-dimensional alternate π-stacked packing of 6-methyl-3-phenylbenzo[*e*][1,2,4]triazine (**I**) along the *b-*axis direction (ellipsoids shown at the 50% probability level). The top two mol­ecules form dimer 1, and the bottom two form dimer 2. Blue dotted lines indicate the closest intra­stack inter­actions (symmetry operation: 1 − *x*, − *y*, 1 − *z*).

The crystal packing of benzotriazine derivatives **I** and **II** exhibits similar supra­molecular features. In both cases, the packing is driven by π–π stacking inter­actions (Meyer *et al.*, 2003 ▸), leading to the formation of one-dimensional (1D) π-stacked columns. In benzotriazine **I**, the mol­ecules are arranged in a centrosymmetric fashion along the stacking direction (Fig. 2 ▸). The center of inversion positions the 3-phenyl and 6-methyl substituents on opposite sides, resulting in the overlap of the triazine ring of one mol­ecule with the benzo-fused ring of its neighbor. The 1D π-stacks in compound **I** exhibit alternating distances between adjacent mol­ecules, forming two distinct dimers with inter­planar separations of 3.257 Å (dimer 1) and 3.304 Å (dimer 2). Within each dimer, the mol­ecules are offset in two directions, giving rise to longitudinal and latitudinal slippage angles of 16.1 and 17.5°, respectively, in dimer 1, and 1.6 and 19.2° in dimer 2. The short inter­planar distances along the 1D alternating chain give rise to a network of short contacts (Fig. 2 ▸), including N⋯C [*d* = 3.255 (1)–3.429 (2) Å], C⋯C [*d* = 3.288 (1)–3.507 (2) Å], and C—H⋯C [*d* = 2.948 Å, angle = 158.5°] inter­actions.

**Figure 3:**
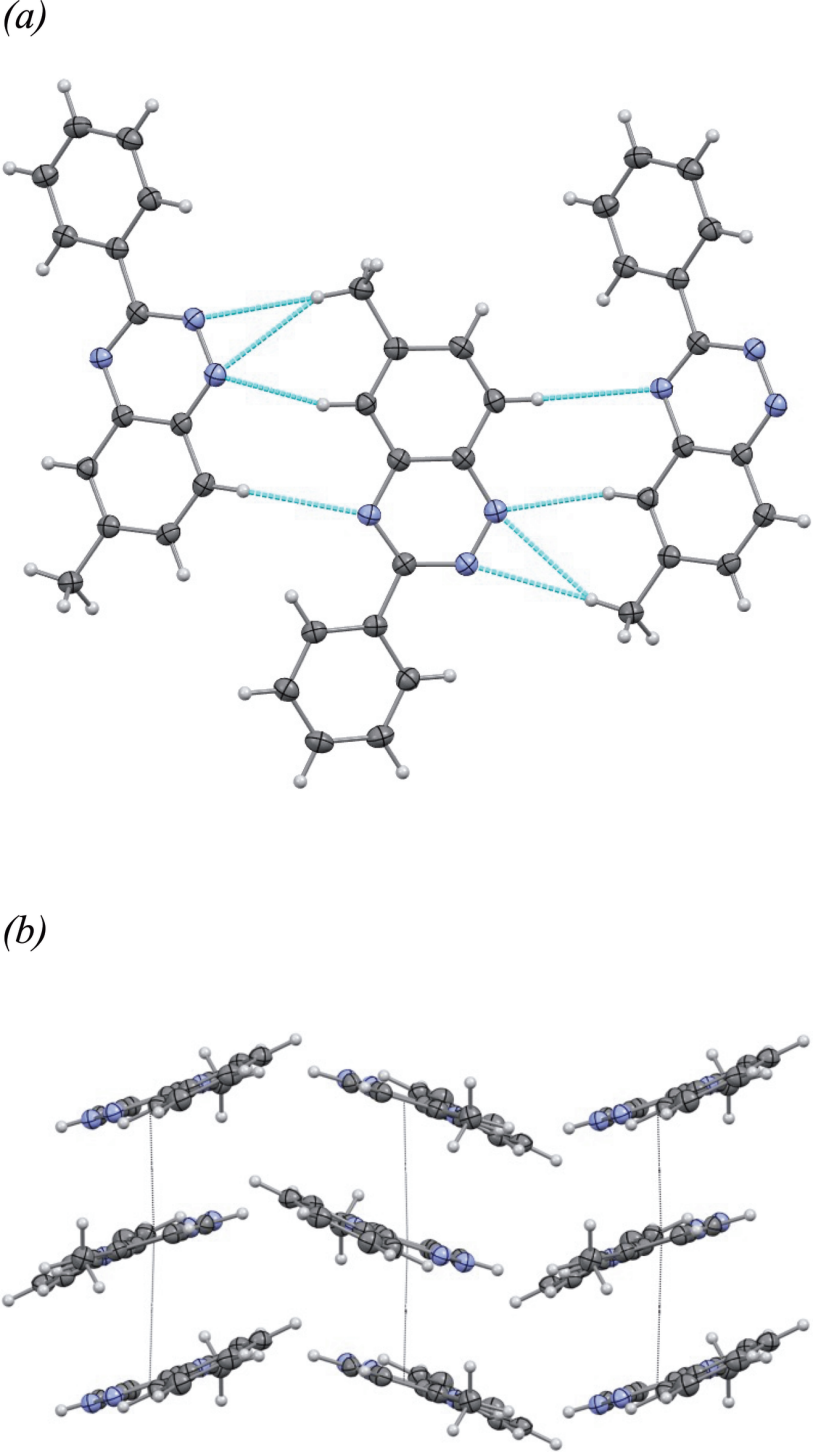
(*a*) One-dimensional chains of **I** running along the *a* axis-direction showing in blue dots the short directional hydrogen-bonding contacts (symmetry code: −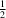 + *x*, 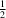 − *y*, 1 − *z*); (*b*) wave-like two-dimensional sheets in the *ab* plane (ellipsoids shown at the 50% probability level).

Mol­ecules of **I** are connected in a head-to-tail fashion, forming chains that extend along the *a*-axis direction (Fig. 3 ▸*a*). Within these chains, the mol­ecules are linked by a series of short directional hydrogen-bonding contacts, including a bifurcated C—H⋯N inter­action [*d* = 2.81 and 2.83 Å; angles = 159 and 149°, respectively], as well as two additional C—H⋯N inter­actions (*d* = 2.49 Å, angle = 151°; *d* = 2.78 Å, angle = 157°). These chains run parallel along the *b*-axis direction, forming closely packed, wave-like two-dimensional sheets in the *ab* plane, with no voids (Fig. 3 ▸*b*). The numerical details of the hydrogen bonds are listed in Table 2 ▸.

**Figure 4:**
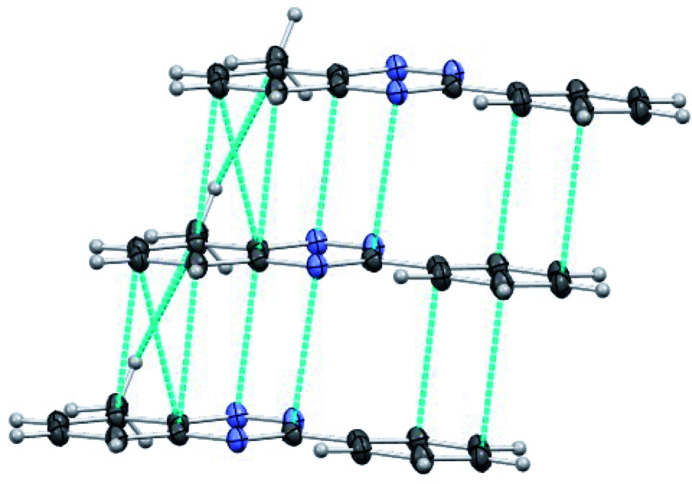
One-dimensional regular π-stacked packing of 8-methyl-3-phenyl­benzo[*e*][1,2,4]triazine (**II**) along the *a-*axis direction (ellipsoids shown at the 50% probability level). Blue dotted lines indicate the closest intra­stack inter­actions (symmetry operation: − 1 + *x*, *y*, *z*).

In benzotriazine **II**, the mol­ecules pack along the *a*-axis direction forming a one-dimensional (1D) π-stacked column, with a regular inter­planar spacing of 3.366 Å, indicative of a well-ordered π-stack (Fig. 4 ▸). Within this π-stack, the mol­ecules are arranged in a translational manner with significant overlap over the benzotriazine ring, although they are slipped relative to each other along both the longitudinal and latitudinal directions, with slip angles of 25.4 and 16.2°, respectively. The short inter­planar distance of 3.366 Å facilitates a network of close inter­molecular π–π stacking contacts, including N⋯C [*d* = 3.378 (2)–3.411 (2) Å], C⋯C [*d* = 3.404 (2)–3.506 (2) Å], and C—H⋯C (*d* = 2.872 Å, angle = 171°; *d* = 2.97 Å, angle = 142°) inter­actions (Fig. 4 ▸).

**Figure 5:**
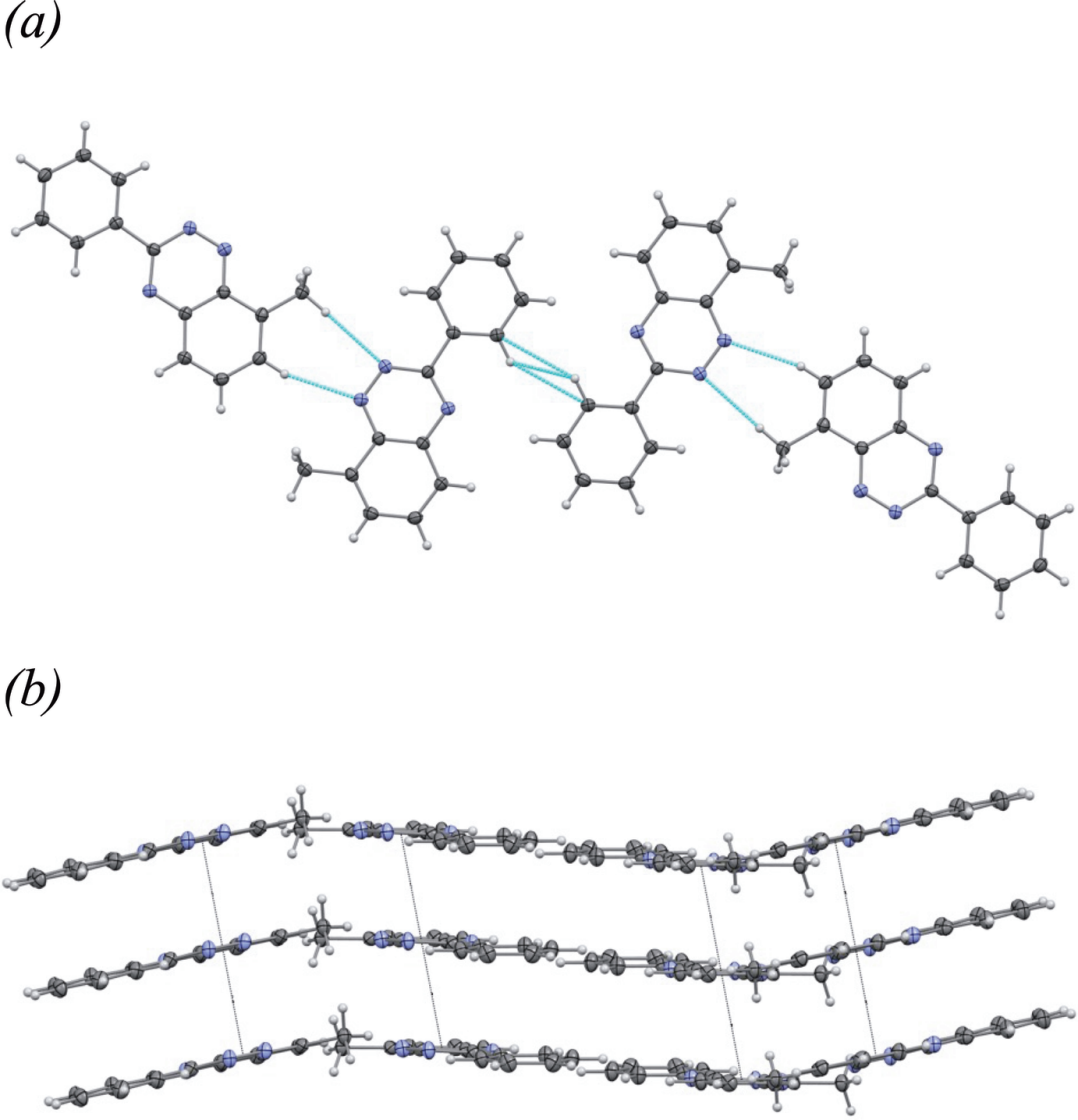
(*a*) One-dimensional chains of **II** running along the *bc* diagonal showing in blue dots the short directional hydrogen-bonding contacts (symmetry operation: −1 + *x*, 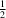 − *y*, −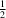 + *z*). Interplanar close contacts excluded for clarity, centroid–centroid distances shown. (*b*) Wave-like two-dimensional sheets in the *ab* plane (ellipsoids shown at the 50% probability level).

Mol­ecules of **II** are connected along the *bc* diagonal to form chains (Fig. 5 ▸*a*). Within these chains, the mol­ecules are linked by a series of short directional hydrogen-bonding contacts, including C—H^·^··N (*d* = 2.57 Å, angle = 161°; *d* = 2.61 Å, angle = 169°) and C—H⋯C (*d* = 2.921 Å, angle = 141°) inter­actions. These chains run parallel along the *bc* diagonal, forming closely packed, wave-like two-dimensional sheets in the *ab* plane, with no voids (Fig. 5 ▸*b*). The numerical details of the intermolecular close contacts are listed in Table 3 ▸

## 4. Database survey

A search of Cambridge Structural Database (CSD, Groom *et al.*, 2016 ▸) of the benzo[*e*][1,2,4]triazine moiety yielded six organic compounds. The first two: 3-phenyl­benzo[*e*][1,2,4]triazine (CCDC GOFDIR, refcode 1873561; Bodzioch *et al.*, 2019 ▸) and 5,7-dimethyl-3-phenyl­benzo[*e*][1,2,4]triazine (CCDC NIFFIR, refcode 129742; Nicolo *et al.*, 1998 ▸) are closely related to the title compounds differing on the number and position of methyl substituents, zero and two, respectively. Their mol­ecular structures show bond lengths and angle patterns indicative of extended conjugated aromatic systems, similar to **I** and **II**. The phenyl and benzotriazine rings are almost planar with 5.70° deviation for GODFIR (Bodzioch *et al.* 2019 ▸) and 2.12° for NIFFIR (Nicolo *et al.*, 1998 ▸), suggesting that number and position of methyls contribute to the rigidity increase. Both GOFDIR and NIFFIR have centrosymmetric arrangements governed by parallel π–π stacking inter­actions. In GODFIR columns are held together by inter­actions between alternating phenyl and benzotriazine moieties, with intra­stack distances of 3.403 Å. Two sets of C⋯C close contacts, with alternating values of 3.396 and 3.382 Å are observed. The neighboring columns are orthogonal with inter­planar angles of 82.36° and inter­stack N⋯H—C hydrogen bonds at 2.61 Å. By contrast in NIFFIR the π–π stacking inter­actions are occurring between benzotriazine cores within dimers with centroid–centroid distance of 3.818 Å. The dimers are held together in ribbons along the *b*-axis direction, with close H⋯H contacts that are close to the sum of van der Waals radii at 2.338 Å, which may be the result of spatial constraints.

The mol­ecular structures of the other four compounds (Kaszynski *et al.*, 2017 ▸): 2-(benzyl­oxycarbon­yl)-1,3-diphenyl-1,2-di­hydro­benzo[*e*][1,2,4]triazine (CCDC HATRAY, CSD refcode 1522809) and 2-(benzyl­oxycarbon­yl)-1-(2-meth­oxy­phen­yl)-3-phenyl-1,2-di­hydro­benzo[*e*][1,2,4]triazine (CCDC HATREC, CSD refcode 1522810) reveal that the N2 atom lies approximately 0.8 Å out of the plane of the 3-phenyl­benzo[*e*][1,2,4]triazine system. This distortion alleviates unfavorable conjugation within the fused ring system and minimizes lone pair inter­actions between the adjacent N1 and N2 atoms. As a result, the two hydrazine substituents, phenyl and benzyl­oxycarbonyl, adopt an anti-orientation relative to each other. The pyramidalization of nitro­gen atoms was found to be more pronounced for N1, effectively preventing conjugation with the aromatic system, while N2 was partially conjugated with the adjacent carbonyl group. Additionally, the steric bulk of both substituents reinforces the rigidity of the mol­ecular conformation, rendering N1 a stable stereocenter.

By contrast, the two isomers 4-(benzyl­oxycarbon­yl)-1,3-diphenyl-1,2-di­hydro­benzo[*e*][1,2,4]triazine (CSD HATQUR, CCDC 1522808) and 4-(benzyl­oxycarbon­yl)-1,3-diphenyl-1,2-di­hydro­benzo[*e*][1,2,4]triazine (CSD HATQOL, CCDC 1522807) adopt a boat conformation, with the planes of the two fused rings forming an angle of 37°. This geometry facilitates improved orbital overlap between the lone pair on N4 and adjacent π* or σ* orbitals. Notably, N1 remains pyramidalized in these structures; however, its stereochemical stability is diminished due to the planarity of N2 and the absence of a second bulky substituent, such as a benzyl­oxycarbonyl group, on N2.

The unit cells of HATRAY, HATQUR, and HATQOL each contain two symmetry-related (inverted) mol­ecules, whereas the unit cell of HATREC contains four. Additionally, water mol­ecules are present in the unit cell of HATRAY. In terms of crystal packing, dimers are formed through close contacts such as CH_2_⋯CO inter­actions in HATQUR and H⋯H inter­actions in HATQOL while in HATREC, mol­ecular chains are stabilized by CO⋯OCH_3_ inter­actions.

## 5. Synthesis and characterization of benzo[*e*][1,2,4]triazines I and II

**Figure 6:**
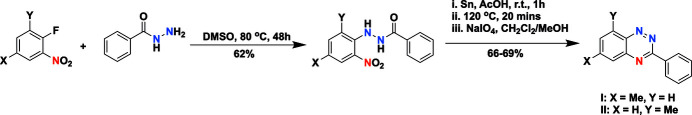
Synthesis of 6-methyl (**I**) and 8-methyl (**II**) 3-phenyl­benzo[*e*][1,2,4]triazines *via* hydrazide formation, reductive cyclo­dehydration, and oxidative aromatization.

Benzotriazines **I** and **II** were synthesized from equimolar amounts of either 1-fluoro-4-methyl-2-nitro­benzene or 2-fluoro-3-nitro­toluene and benzhydrazide, which were reacted in dry DMSO at 33 K for 48 h to afford the corresponding *N*′-(nitro­phen­yl)benzhydrazides in 62% yield after aqueous work-up and recrystallization (Fig. 6 ▸). The hydrazides were then reduced with tin powder in glacial acetic acid at room temperature, followed by brief heating at 393 K to promote cyclo­dehydration and generate the respective di­hydro inter­mediates. Subsequent oxidative aromatization with sodium periodate in a 1:1 mixture of methanol and methyl­ene chloride afforded the target benzo[*e*][1,2,4]triazines, which were purified by silica gel chromatography and recrystallization from ethanol. The 6-methyl (**I**) and 8-methyl (**II**) regioisomers were obtained as yellow solids in 64% and 62% overall yield, respectively, over the three-step sequence. Structural identity and purity were confirmed by NMR, HRMS, and EI-MS.

**Experimental**

Reagents, materials, and solvents were purchased from Sigma Aldrich and used without further purification. ^1^H and ^13^C NMR spectra were recorded on a Bruker Avance 400 MHz spectrometer using the solvent peak as inter­nal reference, with chemical shifts expressed in ppm. The HRMS and fragmentation pattern was collected on Water Xevo G2-XS QTof mass spectrometer with a flow injection method at 0.2 ml/min 95% methanol/5% water, EI method in ion positive mode. Melting points were determined using a MelTemp apparatus.

***N*****′-(4-methyl-2-nitro­phen­yl)benzhydrazide:** A solution of 1-fluoro-4-methyl-2-nitro­benzene (3.10 g, 20.0 mmol) and benzhydrazide (2.72 g, 20.0 mmol) in dry DMSO (10 mL) was stirred at 353 K for 2 days. After cooling, AcOEt (100 mL) followed by H_2_O (150 mL) were added to the reaction mixture and the organic layer was separated. The aqueous layer was extracted twice with small portions of AcOEt. The combined organic layers were dried (Na_2_SO_4_), the solvent was evaporated, and the solid residue was crystallized (ethanol) giving 3.34 g (62% yield) of the hydrazide as yellow crystals: m.p. = 436–438 K; ^1^H NMR (CDCl_3_, 400 MHz) δ 9.03 (*s*, 1H), 8.06 (*s*, 1H), 8.01 (*s*, 1H), 7.89 (*d*, *J* = 7.4 Hz, 2H), 7.61 (*m*, 1H), 7.52 (*m*, 2H), 7.32 (*d*, *J* = 6.9 Hz, 1H), 7.10 (*d*, *J* = 8.6 Hz, 1H), 2.33 (*s*, 3H); ^13^C NMR (CDCl_3_, 400 MHz) δ167, 143, 137, 133, 132, 129, 127, 126, 115, 114, 20; HRMS (ES+) *m*/*z*: [*M*+H]+ calculated for C_14_H_14_N_3_O_3_: 272.1035, found 272.1039; EI-MS (70 eV): *m*/*z* = 105, 77(100%)

**6-Methyl-3-phenyl­benzo[*****e*****][1,2,4]triazine (I):***N*′-(4-methyl-2-nitro­phen­yl)benzhydrazide (2.71 g, 10.0 mmol) was dissolved in glacial acetic acid (100 mL), Sn powder (4.76 g, 40.0 mmol) was added, and the solution was left stirring vigorously at room temperature for 1 hr. The reaction was then heated at 393 K for 20 min and cooled. AcOEt (150 mL) followed by H_2_O (200 mL) were added, and the resulting biphasic mixture was passed through a layer of Celite. The organic layer was separated, and the aqueous layer was extracted with AcOEt (2×). The combined organic extracts were washed with sat. NaHCO_3_ and dried (Na_2_SO_4_). The solvent was removed, the solid residue was dissolved in a MeOH/CH_2_Cl_2_ mixture (1:1, 40 mL), and solid NaIO_4_ (1.5 equivalent) was added. The mixture was stirred until the initial di­hydro derivative was no longer observed by TLC (about 30 min). Inorganic salts were filtered, solvents were evaporated, and the resulting yellow solid residue was passed through a short SiO_2_ column (CH_2_Cl_2_/hexane, 2:1) giving 1.54 g (69% yield) of 6-methyl-3-phenyl­benzo[*e*][1,2,4]triazine as a yellow solid. Subsequent recrystallization (ethanol) gave 1.42 g (64% yield) of pure product: m.p. 368–369 K; ^1^H NMR (CDCl_3_, 400 MHz) δ 8.77 (*dd*, *J* = 7.6, 2.2 Hz, 2H)) , 8.43 (*d*, *J* = 8.6 Hz, 1H), 7.88 (*s*, 1H), 7.69–7.67 (*m*, 1H), 7.64–7.59 (*m*, 3H), 2.68 (*s*, 3H); ^13^C NMR (CDCl_3_, 400 MHz) δ 160, 147, 145, 141, 136, 133, 131, 130, 129, 128, 127, 22; HRMS (ES+) *m*/*z*: [*M*+H]+ calculated for C_14_H_12_N_3_: 222.1031, found 222.1023; EI-MS (70 eV): *m*/*z* = 222, 192, 179, 165 (100%), 152, 104, 89, 77.

***N*****′-(6-Methyl-2-nitro­phen­yl)benzhydrazide:** A solution of 2-fluoro-3-nitro­toluene (3.10 g, 20.0 mmol) and benzhydrazide (2.72 g, 20.0 mmol) in dry DMSO (10 mL) was stirred at 353 K for 2 d. After cooling, AcOEt (100 mL) followed by H_2_O (150 mL) were added to the reaction mixture and the organic layer was separated. The aqueous layer was extracted twice with small portions of AcOEt. The combined organic layers were dried (Na_2_SO_4_), the solvent was evaporated, and the solid residue was crystallized (EtOH) giving 3.34 g (62% yield) of the hydrazide as yellow crystals: m.p. 438–439 K; 1H NMR (DMSO, 400 MHz) δ 10.60 (*s*, 1H), 8.24 (*s*, 1H), 7.80 (*d*, *J* = 7.0 Hz, 2H), 7.64 (*d*, *J* = 7.2 Hz, 1H), 7.57 (*t*, *J* = 7.4 Hz, 1H), 7.48 (*t*, *J* = 7.4 Hz, 2H), 7.43 (*d*, *J* = 7.0 Hz, 1H), 6.98 (*t*, *J* = 7.8 Hz, 1H), 2.42 (*s*, 3H); ^13^C NMR (DMSO, 400 MHz) δ 167, 143, 140, 137, 130, 129, 128, 123, 22, 19; HRMS (ES+) *m*/*z*: [*M*+H]+ calculated for C_14_H_14_N_3_O_3_: 272.1035, found 272.1036; EI-MS (70eV): *m*/*z* = 105 (100%), 77(100%)

**8-Methyl-3-phenyl­benzo[*****e*****][1,2,4]triazine:***N*′-(6-methyl-2-nitro­phen­yl)benzhydrazide (2.71 g, 10.0 mmol) was dissolved in glacial acetic acid (100 mL), Sn powder (4.76 g, 40.0 mmol) was added, and the solution was left stirring vigorously at room temperature for 1 h. The reaction was then heated at 393 K for 20 min and cooled. AcOEt (150 mL) followed by H_2_O (200 mL) were added, and the resulting biphasic mixture was passed through a layer of Celite. The organic layer was separated, and the aqueous layer was extracted with AcOEt (2×). The combined organic extracts were washed with sat. NaHCO_3_ and dried (Na_2_SO_4_). The solvent was removed, the solid residue was dissolved in a MeOH/CH_2_Cl_2_ mixture (1:1, 40 mL), and solid NaIO_4_ (1.5 equivalent) was added. The mixture was stirred until the initial di­hydro derivative was no longer observed by TLC (about 30 min). Inorganic salts were filtered, solvents were evaporated, and the resulting yellow solid residue was passed through a short SiO_2_ column (CH_2_Cl_2_/hexane, 2:1) giving 1.46 g (66% yield) of 8-methyl-3-phenyl­benzo[*e]*[1,2,4]triazine as a yellow solid. Subsequent recrystallization (EtOH) gave 1.38 g (62% yield) of pure product: m.p. 376–377 K; ^1^H NMR (CDCl_3_, 400 MHz) δ 8.80 (*dd*, *J* = 7.7, 2.1 Hz, 2H), 7.95 (*dd*, *J* = 8.9, 1.0 Hz, 1H), 7.88 (*dd*, *J* = 8.6, 6.9 Hz, 1H), 7.67–7.59 (*m*, 4H), 3.08 (*s*, 3H); ^13^C NMR (CDCl_3_, 400 MHz) δ 160, 146, 141, 139, 131, 130, 129, 127, 17; HRMS (ES+) *m*/*z*: [*M*+H]+ calculated for C_14_H_12_N_3_: 222.1031, found 222.1035; EI-MS (70eV): *m*/*z* = 222, 193, 178, 165 (100%), 152, 104, 89, 77.

## 6. Refinement

Crystal data, data collection and structure refinement details are summarized in Table 4 ▸. The diffraction pattern was indexed and the total number of runs and images was based on the strategy calculation DTREK 9.9.9.4 W9RSSI (Pflugrath, 1999 ▸). Hydrogen atom positions were calculated geometrically and refined using a riding model [C—H = 0.95–0.98 Å; *U*_iso_(H) = 1.2*U*_eq_(C) or 1.5*U*_eq_C(meth­yl)].

## Supplementary Material

Crystal structure: contains datablock(s) I, II, srfb6. DOI: 10.1107/S2056989025005882/ev2019sup1.cif

Structure factors: contains datablock(s) I. DOI: 10.1107/S2056989025005882/ev2019Isup2.hkl

Structure factors: contains datablock(s) II. DOI: 10.1107/S2056989025005882/ev2019IIsup3.hkl

CCDC references: 2468228, 2468227

Additional supporting information:  crystallographic information; 3D view; checkCIF report

## Figures and Tables

**Table 1:** 6-Methyl (**I**) and 8-methyl (**II**) 3-phenyl­benzo[*e*][1,2,4]triazine bond lengths and angles (Å, °)

Atoms	Mol­ecule **I**	Mol­ecule **II**	Atoms	Mol­ecule **I**	Mol­ecule **II**
C7—N1	1.374 (1)	1.373 (1)	C8—N2—N1	119.1 (1)	119.08 (9)
N1—N2	1.311 (1)	1.314 (1)	N2—N1—C7	118.9 (1)	119.26 (9)
N2—C8	1.358 (1)	1.360 (1)	N1—C7—N3	125.9 (1)	125.3 (1)
C8—C9	1.421 (2)	1.418 (2)	C7—N3—C9	115.83 (9)	116.01 (9)
C9—N3	1.358 (1)	1.356 (1)	N3—C9—C8	119.5 (1)	119.9 (1)
C7—N3	1.323 (1)	1.326 (1)	C9—C8—N2	120.8 (1)	120.3 (1)
C9—C10	1.414 (2)	1.418 (1)	C13—C8—C9	119.7 (1)	121.1 (1)
C10—C11	1.370 (2)	1.367 (2)	C9—C10—C11	120.3 (1)	118.7 (1)
C11—C12	1.433 (2)	1.420 (2)	C10—C11—C12	119.5 (1)	121.6 (1)
C12—C13	1.362 (2)	1.372 (2)	C11—C12—C13	121.6 (1)	121.7 (1)
C13—C8	1.422 (2)	1.433 (2)	C12—C13—C8	119.2 (1)	117.2 (1)
			C8—C9—C10	119.6 (1)	119.6 (1)

**Table 2:** Hydrogen-bond geometry (Å, °) for **I**[Chem scheme1]

*D*—H⋯*A*	*D*—H	H⋯*A*	*D*⋯*A*	*D*—H⋯*A*
C10—H10⋯N2^i^	0.95	2.49	3.3504 (15)	151
C14—H14*B*⋯N2^i^	0.98	2.83	3.6990 (15)	149
C14—H14*B*⋯N1^i^	0.98	2.81	3.7403 (15)	159
C13—H13⋯N3^ii^	0.95	2.78	3.6771 (14)	157

Symmetry codes: (i) 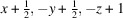; (ii) 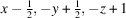.

**Table 3:** Intermolecular close contacts (Å, °) for **II**[Chem scheme1]

*D*—H⋯*A*	*D*—H	H⋯*A*	*D*⋯*A*	*D*—H⋯*A*
C12—H12⋯N2^i^	0.95	2.57	3.4820 (15)	161
C14—H14*B*⋯N1^i^	0.98	2.61	3.5744 (15)	169
C5—H5⋯C5^ii^	0.95	2.92	3.7064 (17)	141

Symmetry codes: (i) 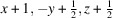; (ii) 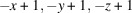.

**Table 4:** Experimental details

	Orthorhombic, *Pbca*	Monoclinic, *P*2_1_/*c*
Crystal data		
Chemical formula	C_14_H_11_N_3_	C_14_H_11_N_3_
*M*_r_	221.26	221.26
Temperature (K)	85	85
*a*, *b*, *c* (Å)	12.2914 (1), 6.9647 (1), 26.4480 (4)	3.8436 (1), 29.6642 (6), 9.6510 (2)
α, β, γ (°)	90, 90, 90	90, 101.196 (2), 90
*V* (Å^3^)	2264.11 (5)	1079.44 (4)
*Z*	8	4
Radiation type	Cu *K*α	Cu *K*α
μ (mm^−1^)	0.63	0.66
Crystal size (mm)	0.24 × 0.22 × 0.12	0.20 × 0.18 × 0.03

Data collection		
Diffractometer	Rigaku MicroMax-007HF	Rigaku MicroMax-007HF
Absorption correction	Multi-scan (*CrysAlis PRO*; Rigaku OD, 2024 ▸)	Multi-scan (*CrysAlis PRO*; Rigaku OD, 2024 ▸)
*T*_min_, *T*_max_	0.832, 1.000	0.825, 1.000
No. of measured, independent and observed [*I* > 2σ(*I*)] reflections	32596, 2110, 2026	16667, 2011, 1809
*R*_int_	0.071	0.084
(sin θ/λ)_max_ (Å^−1^)	0.607	0.606

Refinement		
*R*[*F*^2^ > 2σ(*F*^2^)], *wR*(*F*^2^), *S*	0.055, 0.129, 1.13	0.045, 0.123, 1.11
No. of reflections	2110	2011
No. of parameters	156	156
H-atom treatment	H-atom parameters constrained	H-atom parameters constrained
Δρ_max_, Δρ_min_ (e Å^−3^)	0.29, −0.45	0.20, −0.27

Computer programs: *CrysAlis PRO* (Rigaku OD, 2024 ▸), *SHELXT2018/2* (Sheldrick, 2015*a* ▸), *SHELXL2019/3* (Sheldrick, 2015*b* ▸) and *OLEX2* (Dolomanov *et al.*, 2009 ▸).

## supplementary crystallographic information

### 6-Methyl-3-phenylbenzo[e][1,2,4]triazine (I) Crystal data

**Table:**

C_14_H_11_N_3_	*D*_x_ = 1.298 Mg m^−^^3^
*M**_r_* = 221.26	Cu *K*α radiation, λ = 1.54184 Å
Orthorhombic, *P**b**c**a*	Cell parameters from 18112 reflections
*a* = 12.2914 (1) Å	θ = 4.9–68.9°
*b* = 6.9647 (1) Å	µ = 0.63 mm^−^^1^
*c* = 26.4480 (4) Å	*T* = 85 K
*V* = 2264.11 (5) Å^3^	Needle, yellow
*Z* = 8	0.24 × 0.22 × 0.12 mm
*F*(000) = 928	

### 6-Methyl-3-phenylbenzo[e][1,2,4]triazine (I) Data collection

**Table:**

Rigaku MicroMax-007HF diffractometer	2110 independent reflections
Radiation source: fine-focus sealed X-ray tube, Enhance (Cu) X-ray Source	2026 reflections with *I* > 2σ(*I*)
Graphite monochromator	*R*_int_ = 0.071
ω scans	θ_max_ = 69.4°, θ_min_ = 4.9°
Absorption correction: multi-scan (CrysAlisPro; Rigaku OD, 2024)	*h* = −14→14
*T*_min_ = 0.832, *T*_max_ = 1.000	*k* = −8→8
32596 measured reflections	*l* = −29→31

### 6-Methyl-3-phenylbenzo[e][1,2,4]triazine (I) Refinement

**Table:**

Refinement on *F*^2^	Hydrogen site location: inferred from neighbouring sites
Least-squares matrix: full	H-atom parameters constrained
*R*[*F*^2^ > 2σ(*F*^2^)] = 0.055	*w* = 1/[σ^2^(*F*_o_^2^) + (0.0803*P*)^2^ + 0.4204*P*] where *P* = (*F*_o_^2^ + 2*F*_c_^2^)/3
*wR*(*F*^2^) = 0.129	(Δ/σ)_max_ = 0.008
*S* = 1.13	Δρ_max_ = 0.29 e Å^−^^3^
2110 reflections	Δρ_min_ = −0.45 e Å^−^^3^
156 parameters	Extinction correction: *SHELXL2019/3* (Sheldrick, 2015b), Fc^*^=kFc[1+0.001xFc^2^λ^3^/sin(2θ)]^-1/4^
0 restraints	Extinction coefficient: 0.0197 (12)
Primary atom site location: dual	

### 6-Methyl-3-phenylbenzo[e][1,2,4]triazine (I) Special details

**Table:**

Geometry. All esds (except the esd in the dihedral angle between two l.s. planes) are estimated using the full covariance matrix. The cell esds are taken into account individually in the estimation of esds in distances, angles and torsion angles; correlations between esds in cell parameters are only used when they are defined by crystal symmetry. An approximate (isotropic) treatment of cell esds is used for estimating esds involving l.s. planes.

### 6-Methyl-3-phenylbenzo[e][1,2,4]triazine (I) Fractional atomic coordinates and isotropic or equivalent isotropic displacement parameters (Å^2^)

**Table:**

	*x*	*y*	*z*	*U*_iso_*/*U*_eq_	
N1	0.42769 (8)	0.25701 (14)	0.40469 (4)	0.0252 (3)	
N2	0.38267 (8)	0.20841 (13)	0.44778 (4)	0.0251 (3)	
N3	0.59729 (7)	0.33587 (13)	0.44476 (3)	0.0230 (3)	
C1	0.51948 (10)	0.33532 (17)	0.31007 (4)	0.0277 (3)	
H1	0.450404	0.274567	0.312033	0.033*	
C2	0.56204 (10)	0.38523 (18)	0.26336 (4)	0.0312 (3)	
H2	0.522131	0.357910	0.233454	0.037*	
C3	0.66273 (10)	0.47500 (17)	0.26002 (4)	0.0302 (3)	
H3	0.691704	0.508748	0.227931	0.036*	
C4	0.72093 (10)	0.51524 (16)	0.30386 (4)	0.0280 (3)	
H4	0.789769	0.576694	0.301703	0.034*	
C5	0.67867 (9)	0.46583 (15)	0.35075 (4)	0.0254 (3)	
H5	0.718590	0.494632	0.380555	0.030*	
C6	0.57796 (9)	0.37411 (15)	0.35447 (4)	0.0234 (3)	
C7	0.53352 (9)	0.32035 (15)	0.40467 (4)	0.0230 (3)	
C8	0.44199 (9)	0.22211 (15)	0.49095 (4)	0.0230 (3)	
C9	0.55173 (9)	0.28592 (15)	0.48966 (4)	0.0226 (3)	
C10	0.61263 (9)	0.29357 (15)	0.53498 (4)	0.0239 (3)	
H10	0.685814	0.337367	0.534320	0.029*	
C11	0.56660 (9)	0.23815 (16)	0.57982 (4)	0.0245 (3)	
C12	0.45523 (9)	0.17724 (16)	0.58078 (4)	0.0258 (3)	
H12	0.423284	0.141497	0.612107	0.031*	
C13	0.39409 (9)	0.16935 (16)	0.53788 (4)	0.0253 (3)	
H13	0.320313	0.129061	0.539292	0.030*	
C14	0.63164 (9)	0.23645 (18)	0.62798 (4)	0.0295 (3)	
H14A	0.598802	0.325163	0.652355	0.044*	
H14B	0.706475	0.276657	0.620825	0.044*	
H14C	0.632015	0.106407	0.642129	0.044*	

### 6-Methyl-3-phenylbenzo[e][1,2,4]triazine (I) Atomic displacement parameters (Å^2^)

**Table:**

	*U*^11^	*U*^22^	*U*^33^	*U*^12^	*U*^13^	*U*^23^
N1	0.0255 (5)	0.0243 (5)	0.0259 (5)	0.0006 (4)	0.0005 (4)	0.0003 (4)
N2	0.0251 (5)	0.0240 (5)	0.0263 (6)	0.0004 (4)	0.0003 (4)	0.0002 (4)
N3	0.0252 (5)	0.0216 (5)	0.0222 (5)	0.0010 (4)	0.0007 (4)	0.0003 (4)
C1	0.0280 (6)	0.0271 (6)	0.0281 (6)	0.0005 (4)	−0.0012 (4)	−0.0012 (4)
C2	0.0368 (7)	0.0328 (6)	0.0241 (6)	0.0020 (5)	−0.0042 (5)	−0.0016 (5)
C3	0.0390 (7)	0.0278 (6)	0.0239 (6)	0.0012 (5)	0.0033 (5)	0.0030 (5)
C4	0.0325 (6)	0.0233 (6)	0.0282 (6)	−0.0011 (5)	0.0027 (4)	0.0009 (4)
C5	0.0302 (6)	0.0217 (6)	0.0242 (6)	0.0012 (4)	−0.0018 (4)	−0.0013 (4)
C6	0.0280 (6)	0.0194 (5)	0.0229 (6)	0.0036 (4)	−0.0001 (4)	−0.0003 (4)
C7	0.0257 (6)	0.0183 (5)	0.0250 (6)	0.0016 (4)	−0.0010 (4)	−0.0014 (4)
C8	0.0247 (6)	0.0194 (5)	0.0250 (6)	0.0016 (4)	0.0006 (4)	−0.0003 (4)
C9	0.0249 (6)	0.0184 (5)	0.0243 (6)	0.0017 (4)	0.0009 (4)	0.0000 (4)
C10	0.0232 (6)	0.0235 (6)	0.0252 (6)	0.0010 (4)	0.0008 (4)	0.0003 (4)
C11	0.0276 (6)	0.0215 (6)	0.0245 (6)	0.0033 (4)	0.0006 (4)	−0.0011 (4)
C12	0.0296 (6)	0.0232 (6)	0.0245 (6)	0.0012 (4)	0.0053 (4)	0.0015 (4)
C13	0.0238 (6)	0.0235 (6)	0.0286 (6)	0.0003 (4)	0.0032 (4)	−0.0003 (4)
C14	0.0304 (6)	0.0344 (6)	0.0237 (6)	0.0014 (5)	0.0011 (5)	0.0008 (5)

### 6-Methyl-3-phenylbenzo[e][1,2,4]triazine (I) Geometric parameters (Å, º)

**Table:**

N1—N2	1.3113 (13)	C5—H5	0.9500
N1—C7	1.3735 (14)	C6—C7	1.4835 (15)
N2—C8	1.3580 (14)	C8—C9	1.4206 (16)
N3—C7	1.3231 (14)	C8—C13	1.4221 (15)
N3—C9	1.3583 (14)	C9—C10	1.4141 (15)
C1—C2	1.3858 (16)	C10—C11	1.3695 (16)
C1—C6	1.4032 (15)	C10—H10	0.9500
C1—H1	0.9500	C11—C12	1.4333 (16)
C2—C3	1.3894 (17)	C11—C14	1.5037 (15)
C2—H2	0.9500	C12—C13	1.3621 (16)
C3—C4	1.3909 (15)	C12—H12	0.9500
C3—H3	0.9500	C13—H13	0.9500
C4—C5	1.3877 (15)	C14—H14A	0.9800
C4—H4	0.9500	C14—H14B	0.9800
C5—C6	1.3965 (16)	C14—H14C	0.9800
			
N2—N1—C7	118.88 (9)	N2—C8—C9	120.78 (10)
N1—N2—C8	119.07 (9)	N2—C8—C13	119.56 (10)
C7—N3—C9	115.82 (9)	C9—C8—C13	119.66 (10)
C2—C1—C6	120.29 (11)	N3—C9—C10	120.88 (10)
C2—C1—H1	119.9	N3—C9—C8	119.50 (10)
C6—C1—H1	119.9	C10—C9—C8	119.61 (10)
C1—C2—C3	120.38 (10)	C11—C10—C9	120.31 (10)
C1—C2—H2	119.8	C11—C10—H10	119.8
C3—C2—H2	119.8	C9—C10—H10	119.8
C2—C3—C4	119.72 (10)	C10—C11—C12	119.56 (10)
C2—C3—H3	120.1	C10—C11—C14	121.06 (10)
C4—C3—H3	120.1	C12—C11—C14	119.37 (10)
C5—C4—C3	120.16 (11)	C13—C12—C11	121.61 (10)
C5—C4—H4	119.9	C13—C12—H12	119.2
C3—C4—H4	119.9	C11—C12—H12	119.2
C4—C5—C6	120.55 (10)	C12—C13—C8	119.23 (10)
C4—C5—H5	119.7	C12—C13—H13	120.4
C6—C5—H5	119.7	C8—C13—H13	120.4
C5—C6—C1	118.89 (10)	C11—C14—H14A	109.5
C5—C6—C7	120.33 (10)	C11—C14—H14B	109.5
C1—C6—C7	120.78 (10)	H14A—C14—H14B	109.5
N3—C7—N1	125.93 (10)	C11—C14—H14C	109.5
N3—C7—C6	118.58 (10)	H14A—C14—H14C	109.5
N1—C7—C6	115.48 (9)	H14B—C14—H14C	109.5
			
C7—N1—N2—C8	−0.03 (15)	N1—N2—C8—C9	−0.74 (15)
C6—C1—C2—C3	−0.32 (18)	N1—N2—C8—C13	−179.85 (10)
C1—C2—C3—C4	−0.13 (18)	C7—N3—C9—C10	178.93 (9)
C2—C3—C4—C5	0.05 (18)	C7—N3—C9—C8	−0.01 (15)
C3—C4—C5—C6	0.49 (17)	N2—C8—C9—N3	0.78 (16)
C4—C5—C6—C1	−0.93 (16)	C13—C8—C9—N3	179.88 (9)
C4—C5—C6—C7	179.56 (10)	N2—C8—C9—C10	−178.18 (9)
C2—C1—C6—C5	0.85 (17)	C13—C8—C9—C10	0.92 (16)
C2—C1—C6—C7	−179.65 (10)	N3—C9—C10—C11	−178.33 (10)
C9—N3—C7—N1	−0.82 (16)	C8—C9—C10—C11	0.62 (16)
C9—N3—C7—C6	179.52 (9)	C9—C10—C11—C12	−1.71 (16)
N2—N1—C7—N3	0.87 (16)	C9—C10—C11—C14	177.08 (10)
N2—N1—C7—C6	−179.46 (9)	C10—C11—C12—C13	1.30 (17)
C5—C6—C7—N3	−9.49 (16)	C14—C11—C12—C13	−177.52 (10)
C1—C6—C7—N3	171.02 (10)	C11—C12—C13—C8	0.24 (17)
C5—C6—C7—N1	170.82 (9)	N2—C8—C13—C12	177.78 (9)
C1—C6—C7—N1	−8.68 (15)	C9—C8—C13—C12	−1.33 (16)

### 6-Methyl-3-phenylbenzo[e][1,2,4]triazine (I) Hydrogen-bond geometry (Å, º)

**Table:**

*D*—H···*A*	*D*—H	H···*A*	*D*···*A*	*D*—H···*A*
C10—H10···N2^i^	0.95	2.49	3.3504 (15)	151
C14—H14*B*···N2^i^	0.98	2.83	3.6990 (15)	149
C14—H14*B*···N1^i^	0.98	2.81	3.7403 (15)	159
C13—H13···N3^ii^	0.95	2.78	3.6771 (14)	157

Symmetry codes: (i) *x*+1/2, −*y*+1/2, −*z*+1; (ii) *x*−1/2, −*y*+1/2, −*z*+1.

### 8-Methyl-3-phenylbenzo[e][1,2,4]triazine (II) Crystal data

**Table:**

C_14_H_11_N_3_	*F*(000) = 464
*M**_r_* = 221.26	*D*_x_ = 1.361 Mg m^−^^3^
Monoclinic, *P*2_1_/*c*	Cu *K*α radiation, λ = 1.54184 Å
*a* = 3.8436 (1) Å	Cell parameters from 7484 reflections
*b* = 29.6642 (6) Å	θ = 3.0–68.8°
*c* = 9.6510 (2) Å	µ = 0.66 mm^−^^1^
β = 101.196 (2)°	*T* = 85 K
*V* = 1079.44 (4) Å^3^	Plate, yellow
*Z* = 4	0.20 × 0.18 × 0.03 mm

### 8-Methyl-3-phenylbenzo[e][1,2,4]triazine (II) Data collection

**Table:**

Rigaku MicroMax-007HF diffractometer	2011 independent reflections
Radiation source: fine-focus sealed X-ray tube, Enhance (Cu) X-ray Source	1809 reflections with *I* > 2σ(*I*)
Graphite monochromator	*R*_int_ = 0.084
ω scans	θ_max_ = 69.2°, θ_min_ = 3.0°
Absorption correction: multi-scan (CrysAlisPro; Rigaku OD, 2024)	*h* = −4→4
*T*_min_ = 0.825, *T*_max_ = 1.000	*k* = −35→35
16667 measured reflections	*l* = −11→11

### 8-Methyl-3-phenylbenzo[e][1,2,4]triazine (II) Refinement

**Table:**

Refinement on *F*^2^	Hydrogen site location: inferred from neighbouring sites
Least-squares matrix: full	H-atom parameters constrained
*R*[*F*^2^ > 2σ(*F*^2^)] = 0.045	*w* = 1/[σ^2^(*F*_o_^2^) + (0.0696*P*)^2^ + 0.1404*P*] where *P* = (*F*_o_^2^ + 2*F*_c_^2^)/3
*wR*(*F*^2^) = 0.123	(Δ/σ)_max_ < 0.001
*S* = 1.11	Δρ_max_ = 0.20 e Å^−^^3^
2011 reflections	Δρ_min_ = −0.27 e Å^−^^3^
156 parameters	Extinction correction: *SHELXL2019/3* (Sheldrick, 2015b), Fc^*^=kFc[1+0.001xFc^2^λ^3^/sin(2θ)]^-1/4^
0 restraints	Extinction coefficient: 0.0146 (16)
Primary atom site location: dual	

### 8-Methyl-3-phenylbenzo[e][1,2,4]triazine (II) Special details

**Table:**

Geometry. All esds (except the esd in the dihedral angle between two l.s. planes) are estimated using the full covariance matrix. The cell esds are taken into account individually in the estimation of esds in distances, angles and torsion angles; correlations between esds in cell parameters are only used when they are defined by crystal symmetry. An approximate (isotropic) treatment of cell esds is used for estimating esds involving l.s. planes.
Refinement. The unit cell was refined using CrysAlisPro 1.171.43.130a (Rigaku OD, 2024). The diffraction pattern was indexed and the total number of runs and images was based on the strategy calculation DTREK 9.9.9.4 W9RSSI (Pflugrath, 1999). Empirical absorption correction used spherical harmonics, implemented in SCALE3 ABSPACK algorithm (Dolomanov *et al.*, 2009). The structures were solved and the space group determined by the ShelXT 2018/2 structure solution program using dual methods and refined by full matrix least squares minimisation as implemented in ShelXL 2019/3(Sheldrick, 2015). All non-hydrogen atoms were refined anisotropically.

### 8-Methyl-3-phenylbenzo[e][1,2,4]triazine (II) Fractional atomic coordinates and isotropic or equivalent isotropic displacement parameters (Å^2^)

**Table:**

	*x*	*y*	*z*	*U*_iso_*/*U*_eq_	
N1	0.1239 (2)	0.33776 (3)	0.44411 (10)	0.0228 (3)	
N2	0.2462 (2)	0.30532 (3)	0.53296 (11)	0.0228 (3)	
N3	0.4589 (2)	0.39413 (3)	0.59068 (10)	0.0213 (3)	
C1	−0.1210 (3)	0.40351 (4)	0.23830 (13)	0.0243 (3)	
H1	−0.182424	0.372805	0.219048	0.029*	
C2	−0.2449 (3)	0.43642 (4)	0.13871 (13)	0.0265 (3)	
H2	−0.390752	0.428193	0.051256	0.032*	
C3	−0.1561 (3)	0.48143 (4)	0.16654 (13)	0.0270 (3)	
H3	−0.240374	0.503853	0.097925	0.032*	
C4	0.0550 (3)	0.49356 (4)	0.29421 (13)	0.0276 (3)	
H4	0.115127	0.524306	0.313350	0.033*	
C5	0.1786 (3)	0.46079 (4)	0.39403 (13)	0.0245 (3)	
H5	0.322171	0.469258	0.481710	0.029*	
C6	0.0943 (3)	0.41556 (4)	0.36710 (12)	0.0211 (3)	
C7	0.2367 (3)	0.38118 (4)	0.47472 (12)	0.0204 (3)	
C8	0.4812 (3)	0.31566 (4)	0.65325 (12)	0.0201 (3)	
C9	0.5848 (3)	0.36102 (4)	0.68391 (12)	0.0206 (3)	
C10	0.8219 (3)	0.37133 (4)	0.81177 (12)	0.0232 (3)	
H10	0.892516	0.401564	0.834291	0.028*	
C11	0.9470 (3)	0.33694 (4)	0.90199 (12)	0.0242 (3)	
H11	1.104387	0.343640	0.988216	0.029*	
C12	0.8475 (3)	0.29145 (4)	0.87007 (13)	0.0233 (3)	
H12	0.943361	0.268430	0.934754	0.028*	
C13	0.6166 (3)	0.27987 (4)	0.74827 (12)	0.0211 (3)	
C14	0.5085 (3)	0.23189 (4)	0.71340 (13)	0.0240 (3)	
H14A	0.256052	0.228207	0.715268	0.036*	
H14B	0.648615	0.211719	0.783130	0.036*	
H14C	0.550225	0.224456	0.619041	0.036*	

### 8-Methyl-3-phenylbenzo[e][1,2,4]triazine (II) Atomic displacement parameters (Å^2^)

**Table:**

	*U*^11^	*U*^22^	*U*^33^	*U*^12^	*U*^13^	*U*^23^
N1	0.0254 (5)	0.0174 (5)	0.0235 (6)	0.0009 (4)	−0.0006 (4)	0.0008 (4)
N2	0.0230 (5)	0.0210 (5)	0.0221 (6)	0.0013 (4)	−0.0010 (4)	0.0002 (4)
N3	0.0217 (5)	0.0201 (5)	0.0205 (5)	−0.0003 (4)	0.0000 (4)	−0.0003 (4)
C1	0.0246 (6)	0.0223 (6)	0.0242 (7)	−0.0002 (4)	0.0000 (5)	−0.0022 (5)
C2	0.0260 (6)	0.0285 (7)	0.0220 (7)	0.0024 (5)	−0.0031 (5)	−0.0004 (5)
C3	0.0265 (6)	0.0264 (6)	0.0262 (7)	0.0039 (5)	−0.0001 (5)	0.0065 (5)
C4	0.0288 (6)	0.0217 (6)	0.0297 (7)	−0.0004 (4)	−0.0010 (5)	0.0024 (5)
C5	0.0251 (6)	0.0217 (6)	0.0236 (6)	−0.0006 (4)	−0.0027 (5)	0.0007 (5)
C6	0.0196 (5)	0.0211 (6)	0.0217 (6)	0.0010 (4)	0.0016 (5)	0.0002 (5)
C7	0.0194 (5)	0.0201 (6)	0.0206 (6)	−0.0006 (4)	0.0011 (5)	−0.0027 (5)
C8	0.0193 (5)	0.0204 (6)	0.0202 (6)	0.0005 (4)	0.0025 (5)	−0.0013 (5)
C9	0.0202 (5)	0.0192 (6)	0.0215 (6)	0.0011 (4)	0.0023 (5)	−0.0002 (4)
C10	0.0235 (6)	0.0206 (6)	0.0237 (7)	−0.0012 (4)	−0.0003 (5)	−0.0018 (5)
C11	0.0225 (6)	0.0275 (7)	0.0205 (6)	0.0007 (5)	−0.0015 (5)	−0.0012 (5)
C12	0.0226 (6)	0.0238 (6)	0.0224 (6)	0.0034 (4)	0.0017 (5)	0.0029 (5)
C13	0.0201 (5)	0.0200 (6)	0.0230 (6)	0.0022 (4)	0.0042 (5)	0.0012 (4)
C14	0.0246 (6)	0.0207 (6)	0.0251 (6)	0.0016 (4)	0.0010 (5)	0.0019 (5)

### 8-Methyl-3-phenylbenzo[e][1,2,4]triazine (II) Geometric parameters (Å, º)

**Table:**

N1—N2	1.3137 (13)	C5—C6	1.3926 (15)
N1—C7	1.3728 (14)	C6—C7	1.4824 (15)
N2—C8	1.3604 (15)	C8—C9	1.4182 (16)
N3—C7	1.3261 (15)	C8—C13	1.4332 (15)
N3—C9	1.3562 (15)	C9—C10	1.4177 (15)
C1—H1	0.9500	C10—H10	0.9500
C1—C2	1.3878 (16)	C10—C11	1.3664 (16)
C1—C6	1.3996 (16)	C11—H11	0.9500
C2—H2	0.9500	C11—C12	1.4201 (16)
C2—C3	1.3913 (17)	C12—H12	0.9500
C3—H3	0.9500	C12—C13	1.3719 (16)
C3—C4	1.3850 (17)	C13—C14	1.5023 (15)
C4—H4	0.9500	C14—H14A	0.9800
C4—C5	1.3861 (16)	C14—H14B	0.9800
C5—H5	0.9500	C14—H14C	0.9800
			
N2—N1—C7	119.26 (9)	N2—C8—C9	120.34 (10)
N1—N2—C8	119.08 (9)	N2—C8—C13	118.52 (10)
C7—N3—C9	116.00 (10)	C9—C8—C13	121.14 (10)
C2—C1—H1	120.0	N3—C9—C8	119.95 (10)
C2—C1—C6	119.97 (11)	N3—C9—C10	120.50 (10)
C6—C1—H1	120.0	C10—C9—C8	119.55 (10)
C1—C2—H2	119.9	C9—C10—H10	120.6
C1—C2—C3	120.21 (11)	C11—C10—C9	118.74 (10)
C3—C2—H2	119.9	C11—C10—H10	120.6
C2—C3—H3	120.0	C10—C11—H11	119.2
C4—C3—C2	120.03 (11)	C10—C11—C12	121.64 (10)
C4—C3—H3	120.0	C12—C11—H11	119.2
C3—C4—H4	120.0	C11—C12—H12	119.1
C3—C4—C5	119.90 (11)	C13—C12—C11	121.74 (11)
C5—C4—H4	120.0	C13—C12—H12	119.1
C4—C5—H5	119.7	C8—C13—C14	120.54 (10)
C4—C5—C6	120.68 (11)	C12—C13—C8	117.18 (10)
C6—C5—H5	119.7	C12—C13—C14	122.28 (10)
C1—C6—C7	121.38 (10)	C13—C14—H14A	109.5
C5—C6—C1	119.20 (11)	C13—C14—H14B	109.5
C5—C6—C7	119.42 (10)	C13—C14—H14C	109.5
N1—C7—C6	115.88 (10)	H14A—C14—H14B	109.5
N3—C7—N1	125.33 (10)	H14A—C14—H14C	109.5
N3—C7—C6	118.79 (10)	H14B—C14—H14C	109.5
			
N1—N2—C8—C9	1.70 (17)	C5—C6—C7—N1	176.08 (10)
N1—N2—C8—C13	−179.08 (9)	C5—C6—C7—N3	−4.05 (17)
N2—N1—C7—N3	−1.59 (18)	C6—C1—C2—C3	−0.10 (18)
N2—N1—C7—C6	178.27 (9)	C7—N1—N2—C8	0.13 (16)
N2—C8—C9—N3	−2.29 (17)	C7—N3—C9—C8	0.93 (17)
N2—C8—C9—C10	178.14 (10)	C7—N3—C9—C10	−179.51 (10)
N2—C8—C13—C12	−178.70 (10)	C8—C9—C10—C11	0.52 (17)
N2—C8—C13—C14	1.68 (17)	C9—N3—C7—N1	0.99 (18)
N3—C9—C10—C11	−179.05 (10)	C9—N3—C7—C6	−178.87 (9)
C1—C2—C3—C4	−0.32 (19)	C9—C8—C13—C12	0.51 (17)
C1—C6—C7—N1	−4.44 (17)	C9—C8—C13—C14	−179.12 (10)
C1—C6—C7—N3	175.43 (10)	C9—C10—C11—C12	0.52 (18)
C2—C1—C6—C5	0.64 (18)	C10—C11—C12—C13	−1.09 (19)
C2—C1—C6—C7	−178.84 (10)	C11—C12—C13—C8	0.54 (17)
C2—C3—C4—C5	0.18 (19)	C11—C12—C13—C14	−179.84 (10)
C3—C4—C5—C6	0.37 (19)	C13—C8—C9—N3	178.52 (10)
C4—C5—C6—C1	−0.78 (19)	C13—C8—C9—C10	−1.05 (18)
C4—C5—C6—C7	178.71 (11)		

### 8-Methyl-3-phenylbenzo[e][1,2,4]triazine (II) Hydrogen-bond geometry (Å, º)

**Table:**

*D*—H···*A*	*D*—H	H···*A*	*D*···*A*	*D*—H···*A*
C12—H12···N2^i^	0.95	2.57	3.4820 (15)	161
C14—H14*B*···N1^i^	0.98	2.61	3.5744 (15)	169
C5—H5···C5^ii^	0.95	2.92	3.7064 (17)	141

Symmetry codes: (i) *x*+1, −*y*+1/2, *z*+1/2; (ii) −*x*+1, −*y*+1, −*z*+1.
